# Seasonality of Water Chemistry, Carbonate Production, and Biometric Features of Two Species of *Chara* in a Shallow Clear Water Lake

**DOI:** 10.1155/2014/167631

**Published:** 2014-10-21

**Authors:** Andrzej Pukacz, Mariusz Pełechaty, Marcin Frankowski, Artur Kowalski, Kinga Zwijacz-Koszałka

**Affiliations:** ^1^Polish-German Research Institute, Collegium Polonicum, Kościuszki 1, 69-100 Słubice, Poland; ^2^Department of Hydrobiology, Faculty of Biology, Adam Mickiewicz University in Poznań, Umultowska 89, 61-614 Poznań, Poland; ^3^Department of Water and Soil Analysis, Faculty of Chemistry, Adam Mickiewicz University in Poznań, Umultowska 89b, 61-614 Poznań, Poland; ^4^Faculty of Biology, Adam Mickiewicz University in Poznań, Umultowska 89, 61-614 Poznań, Poland

## Abstract

The objective of this study was to analyze the temporal variability of biometric features and the carbonate production of two charophytes: *Chara polyacantha* A. Braun and *Chara rudis* A. Braun against the background of the physical-chemical properties of water. The investigation was carried out in a small, mid-forest Lake Jasne (western Poland). It is a polymictic, mesotrophic, hardwater ecosystem dominated by charophyte vegetation. Each month, 10 individuals of each species were characterized in terms of morphometric features, fresh and dry weight, and the percentage of calcium carbonate. Additionally, physical-chemical parameters of the water were studied. The results of physical-chemical analyses indicated similar habitat conditions for both species. Despite smaller dry weight *C. polyacantha* was characterized by greater morphological variability and higher rates of growth and percentage share of calcium carbonate in dry mass than *C. rudis*. The percentage of calcium carbonates in dry mass did not differ significantly between the species and exceeded 60%, reaching the maximum (76% in *C. polyacantha*) in July and August. For both species, distinct correlations between the structure of biomass and morphological features were found. The obtained results show the great importance of charophyte vegetation in carbon cycling and functioning of lake ecosystems.

## 1. Introduction

Charophytesare submerged macroscopic green algae, representing the family Characeae (Charales, Charophyceae, and Chlorophyta). Their equisetum-like body called “thallus” forms neither tissues nor typical organs, such as stems, leaves, roots, or flowers. However, attached to the bottom with delicate rhizoids, they can grow from a few centimetres up to more than one meter high [1–3].

The axis of a charophyte thallus is composed of a number of internodes, nodes, and branchlets. From among all charophytes, species of the genus* Chara* represent the most diversified morphological structure. Most of charophytes of this genus have a complex morphology with additional features: bract cells, cortex, spine cells, and stipulodes which are lacking in representatives of* Nitellopsis*,* Nitella*, and* Tolypella*. Moreover, charophytes produce generative and vegetative organs for reproduction. The first are oogonia (female) and antheridia (male), developing mostly at branchlet nodes. The second are bulbils, developed at rhizoids or axial nodes. All those specific features develop specifically and enhance the recognition of the species [2, 4].

A typical feature for most of charophytes is the intense carbonate encrustation on the thalli surface [5–8]. This is a consequence of removing CO_2_, which can be photosynthetically assimilated from soluble bicarbonates, and converting it into insoluble calcium carbonate [9]. In contrast to vascular plants, encrustations adhere tightly to charophytes' thalli. Hence, charophytes are more effective in calcium carbonate precipitation as compared to vascular submerged plants [5, 6, 10]. Therefore, this group of macrophytes is of key importance in calcium carbonate precipitation within the littoral zone, as it emerges from or is postulated in different papers (e.g., [7, 8, 11, 12]). On the basis of literature reports, calcium carbonate encrustations may constitute over 70% [13] and even up to 86% [14] of charophyte dry mass. Therefore, the value of precipitated carbonates within a dense charophyte meadow can reach 1100 g m^−2^, as stated by Pentecost [14]. According to Apolinarska et al. [12] and Pełechaty et al. [8], the average value of precipitated carbonates can exceed 400 g m^−2^.

It is very important for freshwater ecosystems that charophytes can influence water chemistry ([15] and references quoted therein). Compact charophyte beds can produce significant amounts of biomass. As was assumed by L. Kufel and I. Kufel [7], charophytes can produce more biomass than vascular plants and the average values of dry mass fall within the range of 36 to 500 g m^−2^. The maximum values may be much higher, exceeding 1000 g m^−2^ [8, 14, 15]. Such considerable amounts of biomass affect nutrient cycling, particularly in the small and shallow ecosystems [7, 16]. By stabilizing sediments and incorporating nutrients in their biomass, charophytes limit the growth of other macrophytes and phytoplankton (e.g., [17–20]). In addition to biomass, precipitated carbonates play a significant role in the inactivation of nutrients. The photosynthetically accumulated carbon can be deposited in lake sediments with carbonate encrustations [21]. What is noteworthy is that phosphorus can be coprecipitated with calcite, and thus, as an insoluble fraction, it can be stored in sediments [22–24].

The biomass production and carbonate precipitation by charophytes may be conditioned directly and indirectly by many environmental factors. In natural or less disturbed lakes the growth of charophytes is regulated by the delivery rate of nutrients and carbonates from rocks and soils of the lake catchment [8, 12, 25]. Light limitation, as an indirect effect of high nutrient concentration, is thought to be the most relevant for charophyte occurrence [26–29]. Another very important thing is the morphometry of a lake basin [30, 31]. The variability of these factors may affect the differentiation of species morphology, productivity, and carbonate precipitation rates ([8] and references quoted therein).

The current studies indicate that different charophyte species occurring within the same ecosystem produce similar amounts of carbonate encrustation ([24] and references quoted therein). This indicates that carbonate production is not dependent on charophytes' morphology (e.g., the presence of cortification or bract cells) but is site-specific [15, 27, 32–34]. However, it is worth stressing that the existing data are inconclusive and the knowledge on this subject still needs to be supplemented.

The aim of the presented study was to recognize the month-to-month dynamics of biometric features and carbonate production of two charophyte species (*Chara rudis* A. Braun and* Chara polyacantha* A. Braun) against a background of physicochemical properties of water in a mid-forest clear water lake. We hypothesised that the seasonality of water chemistry is reflected in some of the biometric features, although its dynamic is species specific.

## 2. Materials and Methods

**Figure 1:**
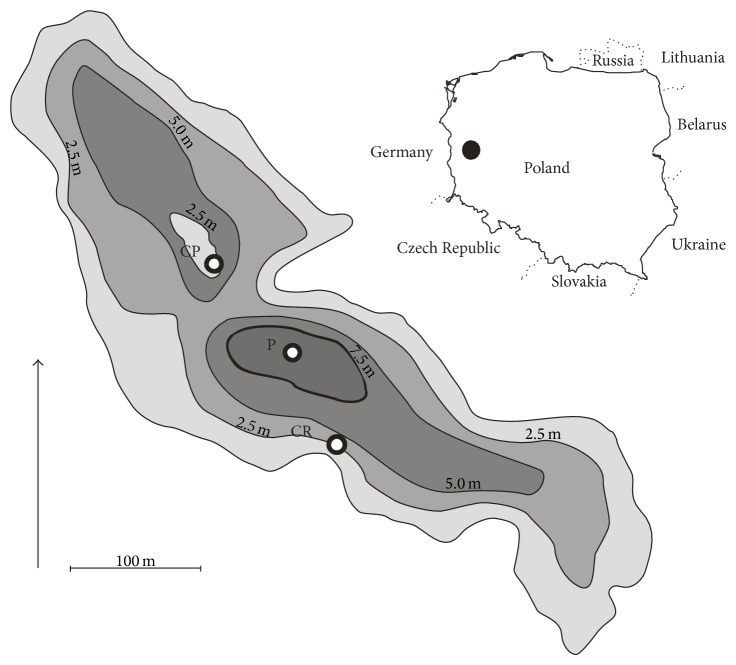
Location of Lake Jasne (mid-western Poland) and distribution of the sampling sites: P: pelagial, CP:* C. polyacantha*, and CR:* C. rudis*.

The study was conducted in a small, mid-forest Lake Jasne (*λ*-15°02′11′′, *ϕ*-52°17′36′′), located in mid-western Poland (Figure 1). The human pressure within the lake is low. Over 90% of the drainage basin is covered by forests (mostly pine forests), which is an important isolating factor due to highly inclined slopes. It belongs to the group of shallow, polymictic lakes, with no fully developed vertical stratification. According to Pelechaty et al. [35], it is one of the clearest lakes within the Lubuskie region. The results of physicochemical analyses of pelagial surface water performed during the vegetation season (month-to-month data, from April to October) evidenced that Lake Jasne was characterized by high transparency and low values of nutrient concentrations, typical for mesotrophic, moderately hard water ecosystems (Table 1).

The field investigations were performed during the 2010 vegetation season (April–October). Each month, at predetermined sites, charophyte individuals were taken for biometric analyses and water samples for physicochemical analyses. Two sample sites were appointed: one in* Chara rudis* bed and second in* Chara polyacantha* bed. The sample sites were selected based on previous observations and investigations (the lake has been investigated regularly from 2005). Both charophyte sites (of ca. 4 m^2^) were at 1.5 m depth, the optimal depth for charophyte growth in Lake Jasne. At each site, charophytes were the dominating species, building dense (covering 100%) monospecific meadows during the whole vegetation season. The plant samples of ca. 100 cm^2^ were harvested manually by diving in order to reduce disturbances within the whole patch. Each month the plant samples were taken from a new place (within the range of a delimited sample site) situated ca. 50 cm from the previous place. The plant material was transported to a laboratory in plastic bags. In the laboratory, 10 individuals were randomly selected from each species sample so as not to damage their structure. Immediately after that, the fresh weight was determined. Next, the following measurements were performed for each individual: axis length, internodium length, axis diameter (measured in the middle of the 3rd internodium from the apex), the number of main branches and side branches, and the number of axis nodes (according to [36]). Additionally, the number of branchlets, the length of branchlets, and the number of branchlet segments were measured in the third whorl of branchlets (counting from the apex), in which the features are properly developed. A stereo microscope Zeiss Stemi DV4 with a photo set Canon PowerShot A640 was used for the measurements. Axio Vision Rel. 4.6 software was applied for the microscopic measurements.

After the measurements, the collected charophytes were dried at 105°C in an electric drier to a constant weight, which was assumed as charophytes' dry weight (D.W.). The contents of organic matter and calcium carbonate (CaCO_3_) were determined by the two-step weight loss on ignition method [37].

Prior to the plant sampling, measurements of basic physicochemical parameters of water were performed, and samples were collected for chemical analyses. Water for these analyses was taken directly from both charophyte sites above (at 1.5 m depth). Additionally, pelagial water was analyzed at each occasion as the reference (the results are presented in Table 1). The following parameters were measured: visibility (only in the pelagial, using a Secchi disc), dissolved oxygen concentration and temperature (using an Elmetron CX-401 portable meter), electrolytic conductivity, and pH (with a Cyber-Scan 200 and 20). After the alkalinity analysis (performed in a laboratory within 6 hours after the sampling), the samples were preserved with CHCl_3_ and kept in a refrigerator (at 4°C) until the remaining chemical analyses were performed.

Total water hardness and Ca^2+^ concentration were determined with the versenate method, while Mg^2+^ concentration was calculated from the difference between total hardness and the concentration of Ca^2+^ ions. The bicarbonate concentration was calculated by multiplying the alkalinity results by 61 g mol^−1^ (the molar mass of HCO_3_^−^). NH_4_^+^ was determined with the spectrophotometric method with Nessler's reagent; NO_2_^−^, with sulphanilic acid and 1-naphthylamine; NO_3_^−^, with the salicylate method; and N-org. with the Kjeldahl method (total nitrogen as a sum of NO_2_^−^, NO_3_^−^, NH_4_^+^, and N-org.). Finally, PO_4_^3−^ was determined with the molybdate method with ascorbic acid as a reducer.

For the interpretation of the obtained results, the Ca/Mg ratio and saturation index (SI) were calculated. For SI, the formula by Kelts and Hsü [38] was applied:

(1) $$\begin{array}{c} \text{SI}=\log\frac{\left[\text{IAP}\right]}{K_{c}}, \end{array}$$

where IAP is Ca^+2^ and CO_3_^−2^ ion activity product and *K*_*c*_ is equilibrium constant for the reaction: CaCO_3_  ⇔  Ca^2+^(aq) + CO_3_^2−^(aq).

Statistical analyses were performed using STATISTICA 10.1 software. Principal Components Analysis (PCA) was performed for the statistical analysis of the physicochemical parameters and morphological data set. Prior to this analysis, the data were subjected to a logarithmic transformation. The Mann-Whitney *U* test (for two groups) and ANOVA by the Kruskal-Wallis H test (for more than 2 groups) were used to determine the significance of differences. *P* < 0.05 was accepted as being statistically sound. Since the number of samples was limited, Spearman rank correlation was applied to test the relationships between charophyte biometric features and the structure of biomass.

Because the speciation forms of nutrients are interrelated, total nitrogen (TN) and total phosphorus (TP) were included in the multivariate analyses. Within the group of morphological features, the number of branches represented both main and site branches.

## 3. Results

### 3.1. Water Properties Variability

**Figure 2:**
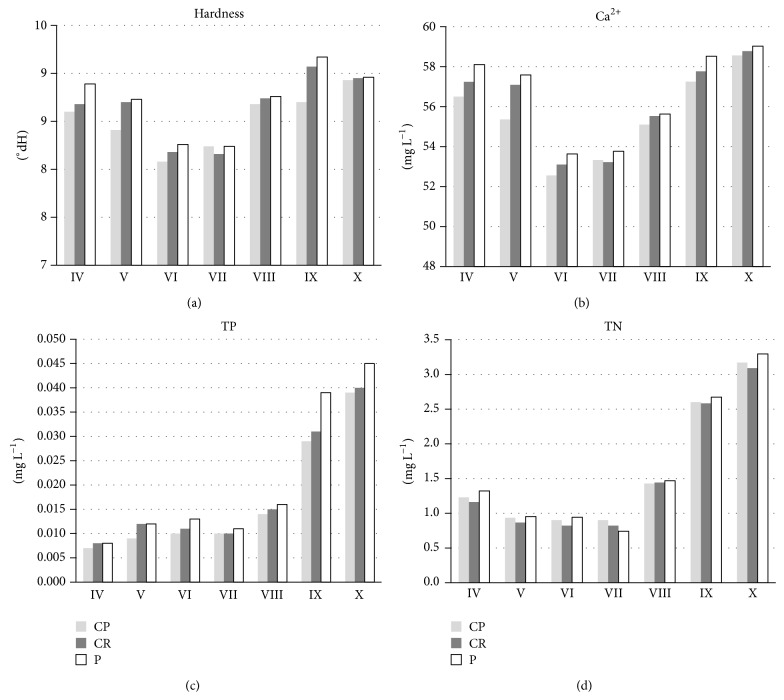
Month-to-month temporal variability of total hardness (a), Ca^2+^ concentration (b), TP: total phosphorus (c), and TN: total nitrogen (d) in water from study sites:* C. polyacantha* site (CP),* C. rudis* site (CR), and pelagial (P). The roman numbers mean the months of study.

No statistically significant differences (Mann-Whitney *U* test, *P* > 0.05) between the littoral sites (CP and CR) and pelagial water (P) in terms of water physicochemistry were found. In all the study sites (both the littoral and the pelagial ones) a similar temporal variability was found. However, for some parameters each month the same pattern of slight differences between the littoral and pelagial water can be observed. The differences were most evident for total hardness (Figure 2(a)), calcium ion concentrations (Figure 2(b)), total phosphorus (Figure 2(c)), and total nitrogen (Figure 2(d)), for which the higher values were observed in pelagic waters.

**Figure 3:**
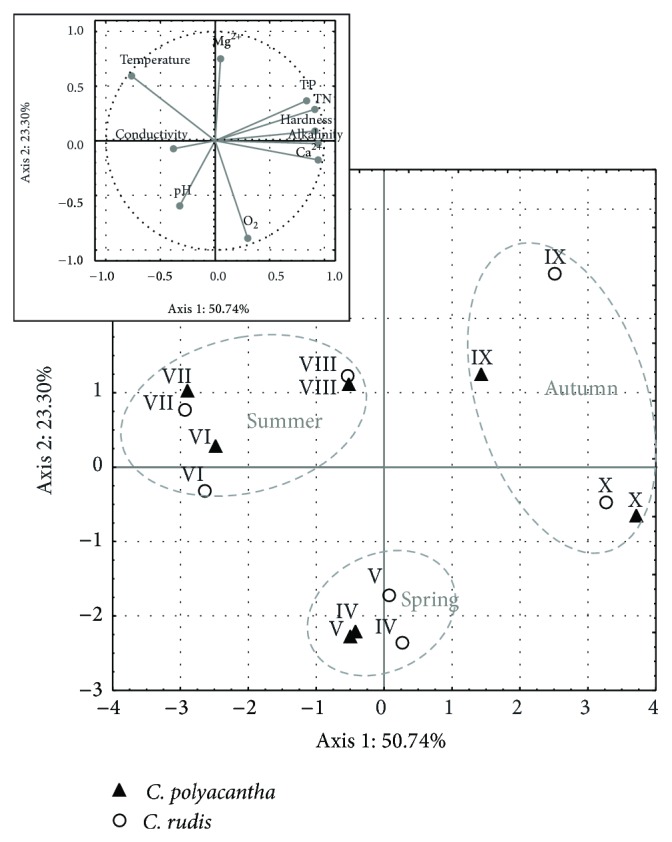
The Principal Components Analysis (PCA) of water properties data gathered during the month-to-month study of* C. polyacantha* and* C. rudis* in Lake Jasne in the 2010 vegetation season. Total phosphorus (TP) and total nitrogen (TN) concentrations represent all the analysed speciation forms of both nutrients. The empty symbols represent* C. rudis* and the filled ones represent* C. polyacantha*. The roman numbers mean the months of study. Dashed grey lines mark the seasons.

No statistically significant differences between both littoral study sites (CP and CR) were found either. To determine the differentiation of water properties within the study sites, PCA for selected physical-chemical parameters was performed. The results of PCA revealed higher differentiation between the study sites only in September (Figure 3). The water in the study sites revealed visible month-to-month variability of most of the physicochemical parameters. The distribution of cases also shows seasonal variation, with a clear division into spring, summer, and autumn. Alkalinity, hardness, and TP, TN, and Ca^2+^ concentrations were correlated most strongly (*r* > 0.75) with the first axis, whereas O_2_ and Mg^2+^ concentrations were correlated most strongly (*r* > 0.75) with the second axis. Both components accounted for almost 75% of the variance observed.

### 3.2. Morphological Variability

**Figure 4:**
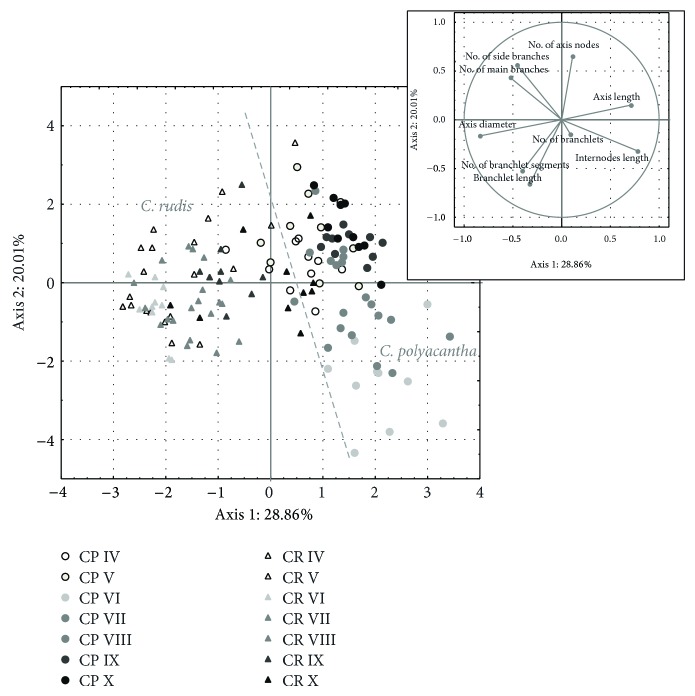
The Principal Components Analysis (PCA) of individuals of both studied species (CP:* C. polyacantha* site, CR:* C. rudis* site)—differentiation across morphological characteristics. The roman numbers mean the months of study. Dashed grey line defines the approximate limit of morphological variability of the species.

To determine the morphological differentiation and define the most differentiating features for investigated species, PCA was performed (Figure 4). The results of this analysis indicated that the two species differed from one another in regard to morphological features, visible as two distinct clusters in the diagram. Generally it can be concluded that* C. polyacantha *is a long and fine species, whereas* C. rudis* is shorter, thicker, and more branchy. The first two main components accounted for almost 50% of the variance observed. Axis length, internode length, axis diameter (correlated with the first axis; *r* > 0.7) and branchlet length, and the number of axis nodes and of side branches (correlated with the second axis; *r* > 0.6) were the features primarily responsible for the variance observed. The results of an additionally performed Mann-Whitney *U* test revealed that these variables also most significantly differed between the two species (*P* < 0.05).

Despite distinct morphological differences, a similar month-to-month variability for both species was observed. In the first half of vegetation season, it was mostly elongation (increase of axis and internodes) that intensified, whereas in the second half, the thalli volume increase (increase of branches number and axis nodes number) prevailed.

Moreover, the distribution of cases shows that* C. polyacantha* appeared to be morphologically more varied than* C. rudis* throughout the investigation time. It was particularly evident for features characterizing elongation (axis length and internode length), which increased considerably in* C. polyacantha* during the peak of vegetation season, whereas in* C. rudis* these features did not vary so much.

### 3.3. Biomass and Calcium Carbonate Production

**Figure 5:**
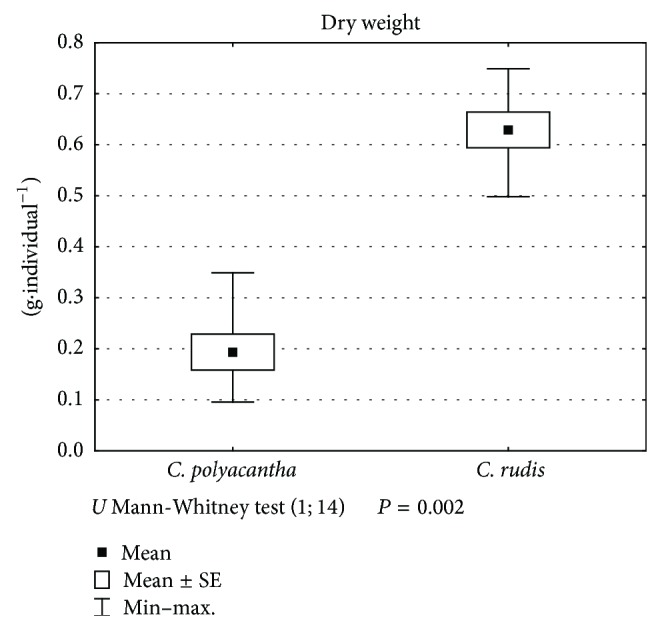
The differentiation between* C. polyacantha* and* C. rudis* against dry weight.

**Figure 6:**
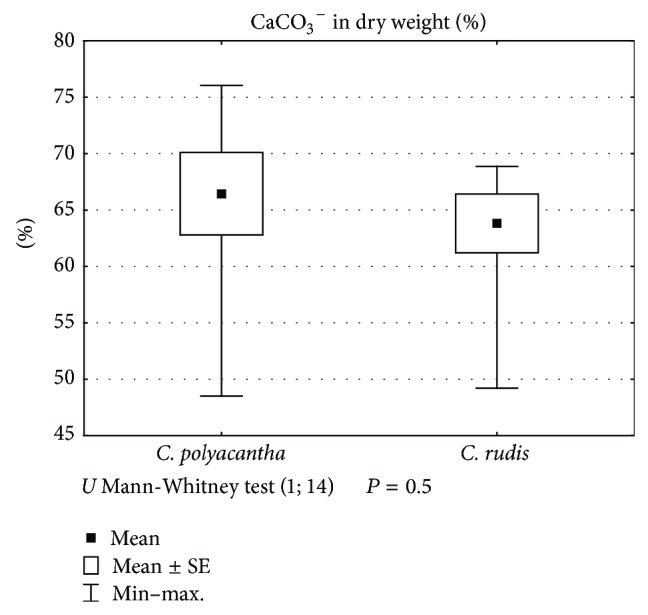
The differentiation between* C. polyacantha* and* C. rudis* against the percentage of CaCO_3_^−^ in dry weight.

The species differed significantly (Mann-Whitney *U* test, *P* < 0.05) in terms of dry weight (Figure 5). Even though the individuals of* C. polyacantha* were significantly higher, the individuals of* C. rudis* produced more than twice as much dry weight. By contrast, the percentage of calcium carbonate encrustations in dry weight (Figure 6) did not differ significantly between the species. Even though the individuals of* C. rudis* produced much more dry weight, the percentage of calcium carbonate was slightly higher in* C. polyacantha*.

**Figure 7:**
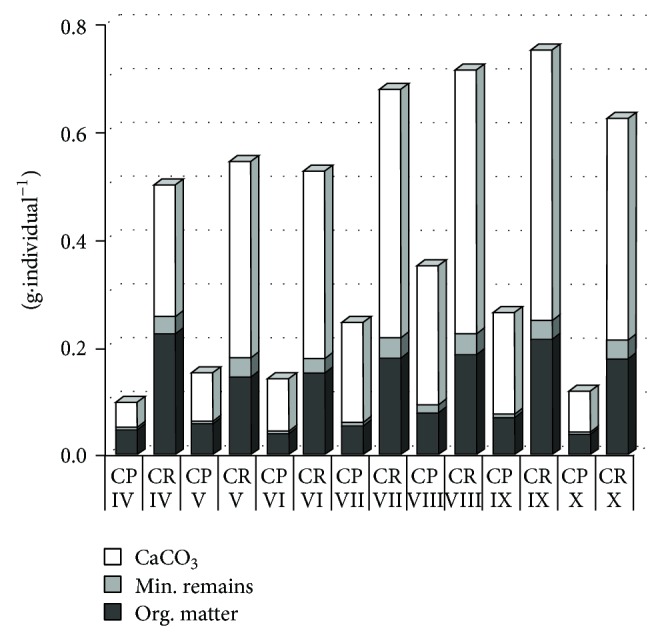
Month-to-month temporal variability of the dry weight structure of* C. polyacantha* (CP) and* C. rudis *(CR). The roman numbers mean the months of study.

The temporal variability of dry and fresh weight had similar patterns for* C. polyacantha* and* C. rudis* (Figure 7). However, the variability was more distinct in* C. polyacantha* (up to 73% in dry weight) than in* C. rudis* (up to 30% in dry weight). For both species the lowest values of biomass were observed in the spring (at the beginning of vegetation season) and in the autumn (at the end of vegetation season). The highest values were in August and were consistent with the general morphological characteristics. In the spring (April–June), the increase of dry and fresh weight was very slight in both species (up to 6% in* C. rudis* and 15% in* C. polyacantha*).The highest increase of dry weight in* C. rudis* was between June and July and slowed down between July and August. In* C. polyacantha*, the highest increase of dry weight was between June and July and between July and August.

The structure of dry weight (organic matter, calcium carbonate, and mineral remains) varied similarly between* C. polyacantha* and* C. rudis* (Figure 7). Both species produced large amounts of carbonate encrustations that constituted over 60% (up to 76% in* C. polyacantha *and up to 69% in* C. rudis*) of dry weight in the whole investigation period. In the second half of vegetation season, the variability of dry weight structure was not so distinct as compared to the spring. The percentage of organic matter in both species was highest in April and lowest in July and August, whereas the opposite trend was evidenced for carbonates—the highest were in July and August and the lowest in April.

The additionally calculated Spearman rank correlations revealed that the dry weight and the percentage of calcium carbonate encrustation in both species are significantly (*P* < 0.05) correlated with some morphological features. In* C. rudis* the dry weight and the percentage of calcium carbonate encrustation were negatively correlated with the number of main branches (*r* = −0.75, *r* = −0.89, resp.) and the number of branchlets (*r* = −0.90, *r* = −0.95, resp.). In* C. polyacantha* the dry weight and the percentage of calcium carbonate encrustation were positively correlated with the axis length (*r* = 0.83, *r* = 0.89, resp.) and the internode length (*r* = 0.71, *r* = 0.79, resp.).

## 4. Discussion

### 4.1. Water Properties Variability

Lake morphometry is one of the main factors affecting the quality and functioning of lake ecosystems [39]. This is particularly evident in the case of shallow lakes where hydrochemical balance is determined by the bilateral biotope-biocoenosis relationship. The similar pattern of temporal variation and no statistically sound differences between the study sites seem to evidence the polymictic character of Lake Jasne.

An important factor regulating habitat conditions in the lake is also the water supply and its exchange rate. It regulates indirectly the amount of minerals and nutrients, thus influencing the primary production [39] and as a result the calcification [17]. As an outlet lake, Lake Jasne is supplied mostly by groundwater of the first aquifer and the precipitation accounts for only 1/4 overall supply. Hence, the water exchange rate for this lake is low, assuming 36% year^−1^. In addition, dense forestation and bushes around the lake and limited human pressure make limited surface runoff during intense rains, thus very limited supply of allochthonous substances (in particular the nutrients). All this promotes stable, mesotrophic conditions favorable to the development of large-area charophyte meadows, reaching 64% of the lake bottom.

No significant differences between the study sites (in particular between the littoral and pelagial ones) could indicate a slight or even no habitat engineering role of charophyte vegetation in Lake Jasne. However, this would be contrary to numerous literature data (e.g., [20, 40, 41]). However, the presented results do not relate to a controlled experiment (e.g., [42, 43]) or the analysis “before and after” (e.g., [6, 44]). In our opinion, no statistically sound differences result from the fact that our studies were carried out in a balanced ecosystem stabilized over the years under the domination of charophyte vegetation [45]. An additional factor contributing to the homogeneity of water is the above-mentioned polymictic character of the lake. Nevertheless, we think that repeatable slight differences between the littoral and pelagial sites observed during the whole investigation period may point to the impact of charophyte vegetation on water properties. The fact that the values of total hardness and calcium ion concentrations were higher in pelagial water testifies to biological decalcification by charophytes (e.g., [39, 46]). The lowest values of total hardness, alkalinity, and concentrations of Ca^2+^ and Mg were observed in August, when the biomass and calcium carbonate participation were the highest, which seems to confirm this phenomenon. It is worth noting that additional statistical analysis revealed no significant correlations between the structure and amount of biomass and the parameters related to hardness (also the pH). It may not only result from the above-mentioned polymictic character of Lake Jasne but also be the consequence of cumulative past uptake, as was suggested by Kufel et al. [24].

The lower values of TP and TN concentrations within charophyte meadows indicate higher primary production and incorporation of these elements in the biomass, as summarized by L. Kufel and I. Kufel [7]. It is in accord with Królikowska [15], who reported lower nutrient concentrations within charophyte meadows in a mesoeutrophic Lake Łuknajno (dominated by charophyte vegetation). Moreover, both parameters decreased in accord with the increase of charophyte biomass, which points to high retention during the vegetation season [47]. With such a high share of charophyte vegetation in littoral, a large pool of nutrients is retained in their biomass. In addition, resuspension of sediments and the release of nutrients into ambient water are limited by dense meadows [41].

### 4.2. Morphological Variability

The morphological differences between* C. polyacantha* and* C. rudis* are in line with the general characteristics of these species known from identification keys (e.g., [2, 48–50]). In our opinion, the features primarily responsible for the obtained variance can be useful as additional traits in identification, particularly having in mind that both species belong to the group of the so-called “tricky species.”

The same patterns of month-to-month changes for specific features seem to confirm similar habitat conditions (the same lake, depth, bottom slopes, exposition, and water chemistry) and population structure (monospecific, 100% cover) in both sites. Some differences concerning different growth rates (higher in* C. polyacantha* than* C. rudis*) in our opinion are just a sign of a species-specific life strategy [51]. A significant growth in the first half of vegetation season (particularly in* C. polyacantha*) and high distribution of values may mean that in very dense meadows charophyte individuals compete against each other for light [33]. Moreover, the fact that, at the end of vegetation season in* C. polyacantha*, the internodes' length decreased and the number of internodes increased proves that the length of charophytes is an outcome of both features.

### 4.3. Biomass and Calcium Carbonate Production

The differences in morphology of the investigated charophyte species were reflected in their biomass. The individuals of* C. rudis*, which are much thicker and more branched and have longer and thicker branchlets, produced much more dry weight than* C. polyacantha* individuals, which have longer thalli. It may suggest that a much more deep-seated* C. polyacantha* meadow will produce a smaller amount of dry mass per square meter than a* C. rudis* meadow.

However, this was not confirmed by investigations in subsequent years, showing that, out of all charophyte species in Lake Jasne,* C. polyacantha* produces the greatest amount of dry weight—up to 2000 g m^−2^. At the same time,* C. rudis* was producing up to 1800 g m^−2^ of dry weight (unpublished data). It may result from the differences in population structure, which may affect the primary production [8, 39]. As is clear from our observations, despite the above-mentioned morphological differences,* C. polyacantha* build much denser meadows than other charophytes in Lake Jasne, which is due to the higher number of individuals per square meter.

Similarly to biomass production, the amount of calcium carbonate encrustation on charophyte thalli is both a direct and indirect effect of primary production [14, 52]. Charophytes are more effective in carbonate precipitation than vascular plants. Still, little is known about the differences between charophyte species in carbonate precipitation, particularly under similar habitat conditions. Our study revealed that* C. rudis* and* C. polyacantha* produced large amounts of carbonate encrustations. There is no literature data about carbonate production by* C. polyacantha*. Nevertheless, values above 70% in the dry weight can be considered as high ([8] and references therein). As was summarized by McConnaughey and Whelan [17] calcification occurs according to “trans” or “cis” model. The “trans” model involves the plant surface separate acid zone, where bicarbonate is protonated, and alkaline zone, dedicated to CaCO_3_ precipitation. This model may lead to intense calcification. The “cis” model does not involve pH zonation (the entire plant surface absorbs CO_2_ and is likely somewhat alkaline) and CaCO_3_ precipitation occurs because CO_2_ uptake by the plant alkalinizes the nearby water. This model may lead to lower ratios of calcification. Thus, the high CaCO_3_ precipitations presented in this study suggest a “trans” physiology.

A previous study in Lake Jasne [53] showed that calcite encrustations deposited on* C. rudis* thalli (individuals taken from compact beds) constituted up to >50% of their dry weight, which was less than in the present study (up to 69%). The difference stems from the fact that, in previous studies, the most photosynthetically active top parts of individuals were analyzed, while in this study we analyzed whole individuals. This may also indicate that the lower parts of charophyte thalli, despite greater shading in a dense meadow, are still active in the precipitation of carbonates. In addition, the lower parts of the plant are the remains from the previous vegetation season [10], as evidenced by the presence of fully developed individuals at the beginning of the study.

It can be concluded, that in alkaline, Ca^2+^-rich waters of Lake Jasne were the optimal conditions to intense calcification [21]. The pH values, ranging between 7.5 and 8.8 in the sample sites, indicate that the dominant form of carbon was HCO_3_^−^ ions. The additionally calculated saturation index SI showed that in both littoral sites the values and their variability were similar. The highest values, ranging from 0.81 to 1.11, were in April and May and then in August. The lowest ones, in turn, were in June and October—0.22 and 0.33, respectively. According to Baumgartner et al. [54], the values of SI that are higher than 0.2 indicate favourable conditions for calcite precipitation. Quite different situation was observed in pelagic water. At the beginning of vegetation season, the SI values were below 0.2 (0.04, −0.03, and −0.3 in April, May, and June, resp.). It is in line with the lower pH values at the beginning of the season (7.9, 7.8, and 7.5 in April, May, and June, resp.), which promote the dissolution of precipitated carbonates and may lead to an increase of HCO_3_^−^ and Ca^2+^ ions [54], which was also found in this study (Figures 2(a) and 2(b)). From July to October, the SI values in pelagial and littoral sites were similar to each other.

The Mg/Ca ratio in all the study sites presented a narrow ratio of 0.04 to 0.06. This indicates, according to Müller et al. [55], that conditions for low Mg carbonate precipitation occurred. Referring to the above-mentioned SI values, it can therefore be concluded that in the whole studied period in the littoral sites conditions promoting the precipitation of low Mg calcite occurred.

Although the last report by Kufel et al. [24] suggests that the production of carbonate encrustation by charophytes is not species-specific, our data do not support this conclusion. The higher percentage of calcium carbonate in* C. polyacantha* dry weight suggests that this species is more efficient at photosynthesis. Due to the obtained physicochemical results and similar site specificity, we exclude the impact of habitat factors, which are considered as the most significant for carbonate precipitation [7, 10]. Another important factor may also be individual features. The precipitation of calcite is dependent upon the presence of nucleation sites [14], which is important particularly when water is supersaturated with calcite [56]. It seems possible that much more spiny* Chara polyacantha* also have a greater active surface which promotes the formation of nucleation sites.

The correlations for* C. rudis* suggest that the increase of individual density (more branched) was not reflected in the increase of dry weight and the percentage of calcium carbonate, whereas the correlations found for* C. polyacantha* showed that an increase in the plant growth (length) was followed by an increase in the dry weight and calcium carbonate production. According to information given by Pełechaty et al. [53], elongation promotes the intensification of photosynthesis. It means that longer* C. polyacantha* individuals growing over a dense meadow are more photosynthetically efficient than* C. rudis *individuals, which building much dense meadows start o limit themselves access to the light.

## Figures and Tables

**Table 1:** Variability of physicochemical parameters in pelagial water in Lake Jasne. Summarized data for the whole study period (April–October). TN: total nitrogen, TP: total phosphorus, and bld: below the limit of detection.

		Minimum	Maximum	Mean ± SD
Temperature	(°C)	8.70	25.30	17.04 ± 6.43
O_2_	(mg L^−1^)	3.96	10.25	6.43 ± 2.17
SD visibility	(m)	2.60	5.80	3.90 ± 1.03
pH		6.80	8.60	7.87 ± 0.59
Conductivity	(*µ*S cm^−1^)	236.00	246.00	241.86 ± 3.72
Alkalinity	(mval L^−1^)	1.60	1.90	1.77 ± 0.13
HCO_3_^−^	(mg L^−1^)	97.60	115.90	108.06 ± 7.65
TP	(mg L^−1^)	0.008	0.045	0.020 ± 0.010
PO_4_^3−^	(mg L^−1^)	bld	bld	bld
TN	(mg L^−1^)	0.74	3.29	1.63 ± 0.98
NO_3_^−^	(mg L^−1^)	0.00	1.53	0.52 ± 0.62
NO_2_^−^	(mg L^−1^)	bld	bld	bld
NH_4_^+^	(mg L^−1^)	0.047	0.382	0.150 ± 0.120
N_org._	(mg L^−1^)	0.59	1.66	0.88 ± 0.41
Ca^2+^	(mg L^−1^)	53.63	59.03	56.61 ± 2.26
Mg^2+^	(mg L^−1^)	2.16	3.47	2.65 ± 0.58
Hardness	(°dH)	8.24	9.19	7.71 ± 0.34
